# Bilateral Optic Perineuritis in Tuberculosis-Immune Reconstitution Inflammatory Syndrome

**DOI:** 10.7759/cureus.27600

**Published:** 2022-08-02

**Authors:** Ismail Fatimah-Halwani, Zulaikha Wahab, Nurul Ain Masnon, Wan-Hazabbah Wan Hitam, Ismail Shatriah, Juhara Haron

**Affiliations:** 1 Department of Ophthalmology and Visual Science, School of Medical Sciences, Health Campus, Universiti Sains Malaysia, Kubang Kerian, MYS; 2 Department of Radiology, School of Medical Sciences, Health Campus, Universiti Sains Malaysia, Kubang Kerian, MYS

**Keywords:** immune reconstitution inflammatory syndrome, idiopathic orbital inflammatory disease, corticosteroids, tuberculosis, optic perineuritis

## Abstract

Optic perineuritis (OPN) in pulmonary tuberculosis (PTB) patients while on anti-tuberculous treatment is rare. It may occur due to tuberculosis-immune reconstitution inflammatory syndrome (TB-IRIS). Visual prognosis is poor if not treated early. We report a rare case of bilateral OPN in an elderly patient on treatment for PTB. A 79-year-old Malay man presented with a painless bilateral blurring of vision for three weeks. He was diagnosed to have PTB and has been on anti-tuberculous treatment for five months. Visual acuity in both eyes was only counting fingers. Optic nerve function tests were significantly reduced bilaterally. Fundoscopy showed bilateral segmental temporal optic disc pallor. Both visual field assessments were constricted. Other infective screenings and tumor markers were negative. Neuro-imaging revealed bilateral OPN involving the intraorbital segment. High-dose intravenous corticosteroid therapy was commenced, followed by slow tapering of oral prednisolone. Anti-tuberculous treatment was continued for a total course of nine months. The left visual acuity improved to 3/60. However, the right eye vision remained poor. His general condition was good.

## Introduction

Tuberculosis-immune reconstitution inflammatory syndrome (TB-IRIS) is an abnormal, overactive immune response against alive or dead Mycobacterium tuberculosis (MTB) that can occur in HIV-infected or, in rare cases, uninfected people [1]. TB-IRIS is the paradoxical worsening or recurrence of pre-existing TB lesions, or the formation of new lesions, among patients on successful anti-TB treatment in the absence of factors that diminish anti-TB efficacy after a particular time period [1]. In the condition of multibacillary illness, the immunopathogenic of IRIS appears to involve a T helper 1-driven immune response [1]. Ocular involvement is about 1%-2% of extrapulmonary manifestation of TB [2]. OPN is a rare inflammatory disorder involving the optic nerve sheath and its adjacent tissues, characterized by visual loss [3]. Although many cases of OPN are isolated and idiopathic, there were few reported cases of secondary OPN related to inflammation or infection [3]. Herein, we report a rare case that presented with bilateral OPN during treatment for PTB.

## Case presentation

A 79-year-old man with underlying good control diabetes mellitus, presented with a gradual painless blurring of vision in both eyes for three weeks. The central vision was mainly affected in the early phase and subsequently, it became generalized. He denied having headaches, nausea, vomiting or fever. There was no history of mouth ulceration, skin lesion, joint pain, or alteration of bowel habits.

He was diagnosed with pulmonary tuberculosis (PTB) five months earlier by the respiratory physician in another center when he presented with chronic cough and loss of weight. The Mantoux test was positive, and the chest x-ray showed pleural effusion with pleural thickening and pleural nodules. Pleural fluid was positive for MTB culture. He was started on an intensive phase of anti-TB treatment, a combination of ethambutol, isoniazid, rifampicin, and pyrazinamide. A month after the intensive phase of anti-TB treatment, the patient developed deranged liver enzymes secondary to pyrazinamide. Eventually, the regime was replaced by ethambutol, isoniazid, rifampicin, and levofloxacin. He was at three months of the maintenance phase of treatment when presenting to our clinic.

**Figure 1:**
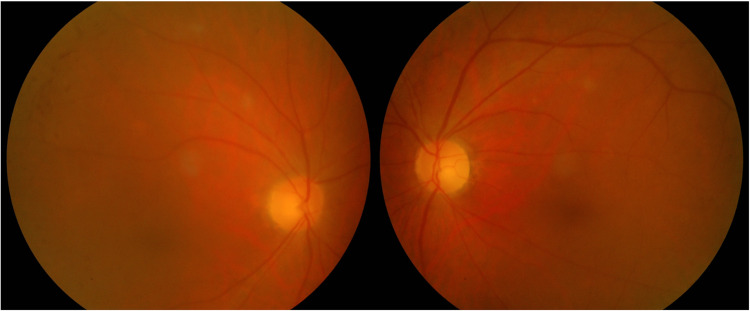
Temporal disc pallor in both eyes.

On examination, the visual acuity (VA) was counting fingers in both eyes. Optic nerve function tests were markedly reduced bilaterally, worst on the right side. Anterior segment examinations were unremarkable except for bilateral immature cataracts. Fundoscopy examination showed bilateral segmental temporal optic disc pallor while the rest of the fundus examinations were normal (Figure 1). The confrontation test showed a bilateral constrictive visual field. Extraocular muscle movements were normal. Blood pressure was 140/70 mmHg, and fasting blood sugar was 8.0 mmol/L. There was no lymphadenopathy. Respiratory examination showed a clear lung on both sides. Neurological and other systemic examination was unremarkable.

**Figure 2:**
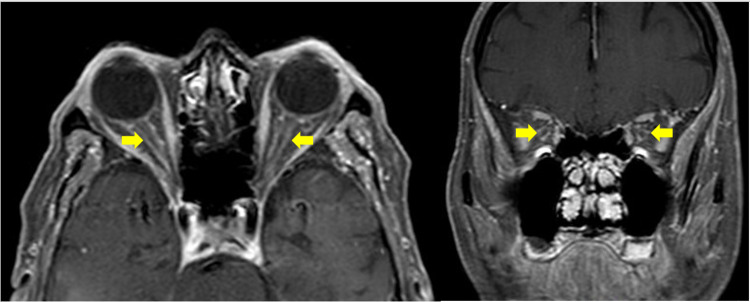
(Left) Axial view of gadolinium fat suppression T1-weighted MR image showed enhancement of bilateral optic nerve sheath “tram track sign” and (right) coronal view of gadolinium fat suppression T1-weighted MR image showed enhancement of bilateral optic nerve sheath “doughnut sign” at the intraorbital segment of optic nerve.

The laboratory results revealed a slightly raised erythrocyte sedimentation rate (ESR) of 42 mm/h. Otherwise the full blood count, C-reactive protein, renal and liver function were within normal limit. Other infective screening including VDRL, HIV, CMV, toxoplasma and herpes, tumor markers and B12 and folate level were normal. Magnetic resonance imaging (MRI) of orbit and brain showed perineural enhancement involving the intraorbital segment of the bilateral optic nerve which represent bilateral optic perineuritis (OPN) (Figure 2). The patient was diagnosed to have bilateral OPN secondary to tuberculosis-immune reconstitution inflammatory syndrome (TB-IRIS).

**Figure 3:**
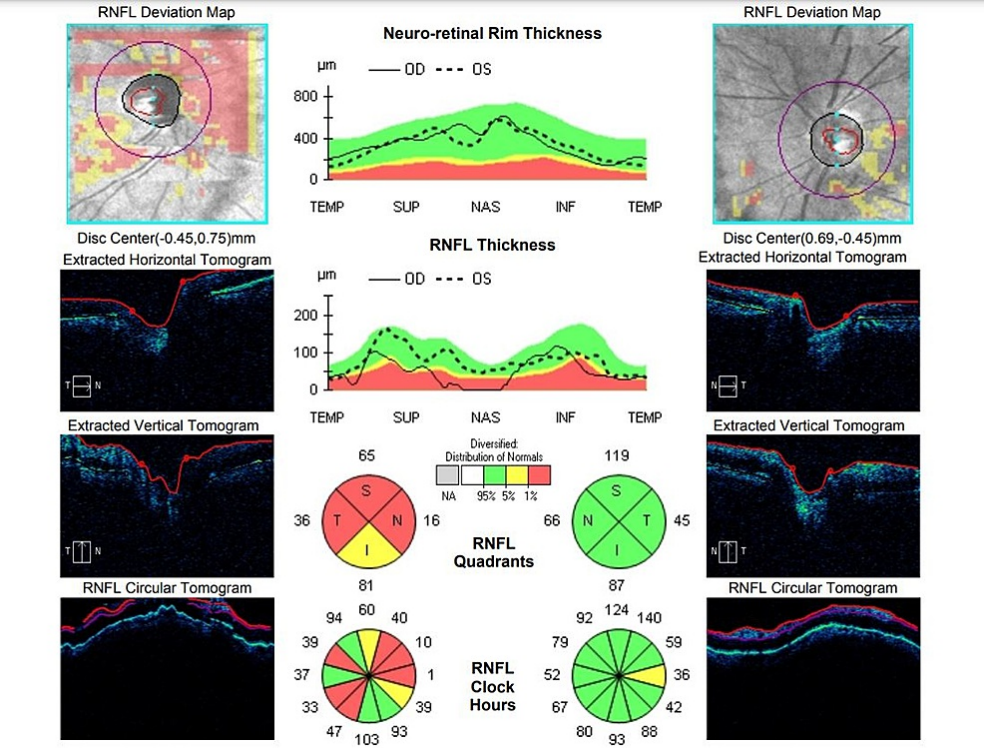
OCT RNFL at four-month follow up showed marked thinning of right RNFL.

The patient was started on intravenous methylprednisolone 250 mg four times per day for five days, followed by oral prednisolone 1 mg/kg/day for two weeks. It was then followed by a tapering dose over three months. Anti-TB treatment was continued for another four months to complete for a total nine-month course. The patient tolerated both the new anti-TB regime and corticosteroids treatment without any serious side effects and was monitored by the respiratory team. On the latest follow up after four months of completed treatment, the patient had a very minimal visual recovery. His VA in the left eye slightly improved to 1/60 while the right eye remained hand movement. Fundoscopy showed bilateral optic disc atrophy. A significant thinning of RNFL in all four quadrants (Figure 3) was observed in the right eye, consistent with the VA findings. 

## Discussion

OPN is a form of idiopathic orbital inflammatory disease, in which the specific target tissue is the optic nerve sheath [4]. OPN is classified into idiopathic OPN or secondary OPN. Idiopathic OPN is a primary syndrome where no underlying causes have been identified whereas secondary OPN is when there is an identifiable systemic disorder [4]. Tuberculosis (TB), Syphilis, Wegener’s granulomatosis, sarcoidosis, Crohn's disease, and leukemia have been reported as a cause of OPN [5-8]. TB is an infectious disease caused by the bacillus MTB. TB is a well-known disease since ancient times and remains one of the leading causes of morbidity and mortality worldwide, with 10 million new incident TB cases in 2017 [9]. The South-East Asia region accounts for 39% of the global burden of TB in terms of incidence [9]. It is estimated that about 3.4 million new cases of TB continue to occur each year in this region [9].

Ocular involvement occurs in about 1% to 2% of patients with TB. In the eye, TB can affect any structures which can be unilateral or bilateral [10]. Ocular involvement can result from hematogenous spread, direct local extension, or as a hypersensitivity response to distant infection [10]. IRIS is a significant immune system reaction to an infectious or non-infectious antigen caused by immunological recovery once immunosuppression is removed. TB-IRIS is therefore an early consequence of antituberculosis treatment or antiretroviral treatment (ART) in MTB-infected individuals with or without HIV coinfection [11]. Although TB-IRIS is well known among HIV-positive patients on ART, it is less frequent and understood in HIV-uninfected patients. TB-IRIS is estimated to affect 2% to 23% of HIV-negative individuals undergoing antituberculosis medication [11]. The diagnosis of bilateral OPN secondary to TB-IRIS was made after we exclude the other possible causes including another infective, inflammatory, infiltrative and nutritional causes.

In OPN, MRI demonstrates a sign of circumferential optic nerve sheath enhancement instead of intraneural enhancement in optic neuritis [12]. The diagnosis is commonly based on a combination of clinical and radiographic findings [12]. This is in keeping with our case where the MRI showed perineural enhancement of the optic nerve bilaterally.

High dose corticosteroids have been reported to successfully treat both idiopathic and secondary OPN without recurrence [1,2,6,13]. However, some cases had relapsed on corticosteroid tapering leading to additional treatment with other immunosuppressants [4]. The prognosis of OPN has been reported to be poor when the initiation of treatment is delayed [13]. This is consistent with our case, as the patient only presented after three weeks of the onset of the symptoms. Our case highlighted that late and chronic presentation with delayed corticosteroids therapy is possibly a factor leading to poor visual prognosis in patients with OPN secondary to TB-IRIS.

## Conclusions

This case is unique as OPN is a rare presentation of TB-IRIS, which developed while the patient was on anti-TB treatment. TB-IRIS is potential sequelae of anti-TB treatment which need to be anticipated and monitored. Early identification of TB-IRIS as a possible sequela of anti-TB treatment may offer early initiation of adjunctive corticosteroid therapy, which may lead to a better visual prognosis.
